# Supplementary material for: Effects of gestational age on blood cortisol and prolactin levels during pregnancy in malaria endemic area
Source: PLoS One. 2024 Nov 4;19(11):e0310372. doi: 10.1371/journal.pone.0310372 (PMC11534236; doi:10.1371/journal.pone.0310372)
Supplement: S1 Table — This table summarize the value of cortisol and prolactin at scheduled visit. (DOCX) [file pone.0310372.s002.docx]

Supporting table 1: Concentration of cortisol and prolactin at scheduled visit and delivered.

|  | Cortisol  ng/ml [IQR] | | Prolactin  ng/ml [IQR] | |
| --- | --- | --- | --- | --- |
| Scheduled visits | Primigravidae | Multigravidae | Primigravidae | Multigravidae |
| Visit 1 | 99.2 [86.4-130.1] | 94.4 [85.9-134.5] | 88.9 [71.5-126.7] | 33.9 [30.4-74.8] |
| Visit 2 | 127.7 [111.7-134.4] | 116.4 [98.8-124.2] | 191.0 [141.3-293.7] | 134.0 [84.6-219.9] |
| Visit 3 | 108.2 [97.6-124.5] | 107.6 [100.2-119.1] | 334.3 [226.0-420.5] | 250.8 [133.9-326.1] |
| Visit 4 | 172.7 [159.9-190.5] | 128.3 [118.8-174.7] | 269.2 [170-359] | 368.0 [285.1-583.5] |
| Visit 5 | 79.3 [50.4-96.5] | 59.2 [47.1-88.2] | 144.4 [111.6-205.0] | 281.0 [165.2-339.6] |
