# Supplementary material for: Effects of gestational age on blood cortisol and prolactin levels during pregnancy in malaria endemic area
Source: PLoS One. 2024 Nov 4;19(11):e0310372. doi: 10.1371/journal.pone.0310372 (PMC11534236; doi:10.1371/journal.pone.0310372)
Supplement: Attachment — Submitted filename: comments.docx [file pone.0310372.s004.docx]

The manuscript investigates the effect of gestation on cortisol and prolactin levels in pregnant women, comparing primigravidae and multigravidae. The study design, methodology, and data analysis are well-described, and the results are presented clearly and concisely. The discussion adequately interprets the findings in the context of existing literature.

Major Comments:

1. Background:
   - The introduction could be strengthened by providing a more focused review of the role of cortisol and prolactin in pregnant women, particularly in relation to immune function and stress response.
   - The concept of "suppression of (some) immune responses" could be further elaborated upon, differentiating between specific types of immune responses and the nuances involved.
2. Methods:
   - The inclusion and exclusion criteria for participants should be clearly stated, especially for the control groups.
   - It would be helpful to explain the rationale behind the chosen time points for sample collection.
   - The description of the laboratory procedures could be expanded to include details such as the manufacturers and specific kits used for the cortisol and prolactin assays.
3. Results:
   - The tables and figures could be improved by adding more descriptive captions and legends.
   - Consider including statistical tests and p-values in the figures directly.
   - It would be helpful to present the results of the correlation analysis between cortisol and prolactin in a separate table.
4. Discussion:
   - The discussion could be strengthened by addressing limitations of the study, such as the relatively small sample size and potential confounding factors.
   - Consider including more references to support the claims made about the roles of cortisol and prolactin in pregnancy.

Minor Comments:

- Consider using a consistent formatting style for references throughout the manuscript.
- Ensure that all abbreviations are defined upon first use.
- Check for typos and grammatical errors.

Specific Points:

- Page 5: Define IPT (immunologic pregnancy test).
- Page 6: Explain how the detection limit of 100 ng/ml for prolactin was adjusted for higher concentrations.
- Page 7: Clarify what is meant by "non-response" in the sample size calculation.
- Page 7: Mention the statistical test used for comparing cortisol and prolactin concentrations between gravidity groups.
- Page 7: Specify the factors included in the LMER model for assessing the interplay between cortisol and prolactin.

Overall, the manuscript presents valuable data on the modulation of cortisol and prolactin levels during pregnancy. Addressing the major and minor comments will further strengthen the manuscript and improve its clarity and impact.
