# Supplementary material for: Effects of gestational age on blood cortisol and prolactin levels during pregnancy in malaria endemic area
Source: PLoS One. 2024 Nov 4;19(11):e0310372. doi: 10.1371/journal.pone.0310372 (PMC11534236; doi:10.1371/journal.pone.0310372)
Supplement: Attachment — Submitted filename: Kiemde et al_Rebuttal_PONE-D-23-33355R1_29052024_Final.docx [file pone.0310372.s005.docx]

**REBUTTAL TO THE POINTS RAISED BY REVIEWERS:**

**Reviewer #2** (No response)

**Reviewer #3**

**General comment**: Dear authors, Many thanks for your research. Here are some comments on your original research.

**RESPONSE:** ***We would like to thank the reviewer for his/her time to review the resubmission of the manuscript. All the comments have been addressed in the review manuscript.***

**Comment 1**: In line 208 and 209 “The detection limit of prolactin with ichroma II is 100ng/ml. In case of concentration over 100ng/ml, ¼ and 1⁄8 dilution of the plasma was done with saline phosphate buffer as recommended” the detection limit is an incorrect phrase. In clinical laboratory, we describe the detection limit as: the lowest concentration that the procedure (laboratory kit) can detect. The highest concentration that the procedure or kit can detect and higher concentrations should be diluted is defined as the linearity range. Therefore, dear authors should correct this definition. Please, refer to kit inset sheet/instruction about the linearity and detection limit complementary descriptions.

**RESPONSE:** ***We would like to thank the reviewer for this observation regarding the detection limit. We have now corrected the manuscript: line 208:*** “The threshold for prolactin quantification with ichroma II is 100ng/ml”.

**Comment 2**: In figures 1 and 2 (box plots), please, correct the p-values decimals. Please, remove extra decimals

**RESPONSE:** ***We would like to thank the reviewer for this observation. We have now arranged the p-values in the figure directly and add a legend to the figure.***

**Comment 3**: In discussion section, I think “the first year of live” is better to be replaced with “the first year of life”.

**RESPONSE:** ***Now we are corrected. Please see line 401 – 403:*** “Nevertheless, their role in the development of new-born immunity and the prevention to infectious disease such as malaria during the first year of life remains unclear”.

**Comment 4**: In discussion section, Lines 411 and 412 “This is an explorative study conducted in pregnant women. The study has been designed to determine the cytotoxicity of NK cells against erythrocytes infected by Plasmodium falciparum.” This phrase is related to which study? If it is related to the current study, I can not see any direct variable related to NK cells and immune system. If this is not related to the current study, please remove it from discussion and abstract and elsewhere in body text. Many thanks with the best wishes

**RESPONSE:** ***We have now deleted the following sentence to the manuscript:*** “The study has been designed to determine the cytotoxicity of NK cells against erythrocytes infected by *Plasmodium falciparum*”.
